# Supplementary material for: Clearance of senescent decidual cells by uterine natural killer cells in cycling human endometrium
Source: eLife. 2017 Dec 11;6:e31274. doi: 10.7554/eLife.31274 (PMC5724991; doi:10.7554/eLife.31274)
Supplement: Supplementary file 1. — Data are related to primary cultures or individual figures as indicated. Primary cultures refer to all biopsies from which EnSCs were isolated and propagated in culture. *Values are presented as mean ±SD. ^Values are presented as median (range). N/A: not applicable. [file elife-31274-supp1.docx]

**Supplementary File 1**

**Table 1. Patient demographics.**

| **Experiment** | **Primary cultures** | **Figure 2B** | **Figure 3D** | **Figure 5A** | **Figure 5B** |
| --- | --- | --- | --- | --- | --- |
| **Number** | 109 | 73 | 308 | 1997 | 20 |
| **Age *** | 36.1 ± 4.2 | 34.7 ± 4.8 | 35.8 ± 4.3 | 35.98 ± 4.4 | 36.6 ± 5.7 |
| **BMI *** | 25.2 ± 4.3 | 25.9 ± 5.5 | 24.4 ± 4.5 | 25.2 ± 4.7 | 26.4 ± 4.5 |
| **Day of cycle ^ (LH+)** | 9 (6-12) | N/A | 8 (6-12) | 8 (5-12) | 8 (6-11) |
