# Supplementary material for: Clearance of senescent decidual cells by uterine natural killer cells in cycling human endometrium
Source: eLife. 2017 Dec 11;6:e31274. doi: 10.7554/eLife.31274 (PMC5724991; doi:10.7554/eLife.31274)
Supplement: Transparent reporting form [file elife-31274-transrepform.docx]

***eLife’s* transparent reporting form**

We encourage authors to provide detailed information *within their submission* to facilitate the interpretation and replication of experiments. Authors can upload supporting documentation to indicate the use of appropriate reporting guidelines for health-related research (see [EQUATOR Network](http://www.equator-network.org/%20)), life science research (see the [BioSharing Information Resource](https://biosharing.org/)), or the [ARRIVE guidelines](http://www.plosbiology.org/article/info:doi/10.1371/journal.pbio.1000412) for reporting work involving animal research. Where applicable, authors should refer to any relevant reporting standards documents in this form.

If you have any questions, please consult our Journal Policies and/or contact us: [editorial@elifesciences.org](mailto:editorial@elifesciences.org).

**Sample-size estimation**

- You should state whether an appropriate sample size was computed when the study was being designed
- You should state the statistical method of sample size computation and any required assumptions
- If no explicit power analysis was used, you should describe how you decided what sample (replicate) size (number) to use

Please outline where this information can be found within the submission (e.g., sections or figure legends), or explain why this information doesn’t apply to your submission:

This study combines *in vitro* analysis on primary cell cultures with *ex vivo* analysis of whole tissue biopsies. It does not compare responses between patient groups. Hence, no formal power calculations were carried out.

For *in vitro* investigations, data were generated from a minimum of 3 independent primary cultures (i.e. biological replicates). The number of biological repeat experiments is stated in figure legends and source data file.

For *ex vivo* analyses, sample size and grouping were dependent on sample availability – this being in part dictated by the day of cycle on which patients attended the Implantation Research Clinic. The number of samples is stated in figure legends, and Supplementary File 1

**Replicates**

- You should report how often each experiment was performed
- You should include a definition of biological versus technical replication
- The data obtained should be provided and sufficient information should be provided to indicate the number of independent biological and/or technical replicates
- If you encountered any outliers, you should describe how these were handled
- Criteria for exclusion/inclusion of data should be clearly stated
- High-throughput sequence data should be uploaded before submission, with a private link for reviewers provided (these are available from both GEO and ArrayExpress)

Please outline where this information can be found within the submission (e.g., sections or figure legends), or explain why this information doesn’t apply to your submission:

Biological replication refers to the number of primary cultures established from different endometrial biopsies. Technical replication refers to the number of repeat measurements in a given sample in response to a given treatment at a given time-point. Technical replication involved duplicate or triplicate repeat measurements, depending on the assay. A minimum of 3 biological replicates were used in each experiment and indicated within figure legends.

No outliers were excluded in the in vitro experiments. In Figures 2B and 5A, to obtain confidence intervals, p16 or uNK scores at each day of the cycle were fitted into a beta distribution with the fitdistr function from the MASS package in R version. The resulting percentile values were smoothed with spline interpolation using the spline function of the basic stats package of R and converted into a heatmap. Details are available in the Materials and Methods section.

Inclusion / exclusion criteria for patient participation are detailed in Materials and Methods section.

**Statistical reporting**

- Statistical analysis methods should be described and justified
- Raw data should be presented in figures whenever informative to do so (typically when N per group is less than 10)
- For each experiment, you should identify the statistical tests used, exact values of N, definitions of center, methods of multiple test correction, and dispersion and precision measures (e.g., mean, median, SD, SEM, confidence intervals; and, for the major substantive results, a measure of effect size (e.g., Pearson's r, Cohen's d)
- Report exact p-values wherever possible alongside the summary statistics and 95% confidence intervals. These should be reported for all key questions and not only when the p-value is less than 0.05.

Please outline where this information can be found within the submission (e.g., sections or figure legends), or explain why this information doesn’t apply to your submission:

All information pertaining statistical reporting (including the number of biological repeats, definitions of center and dispersion, are detailed in individual figure legends and in the source data file. Throughout the study, Kolmogorov-Smirnov test was used to check the distribution of data. Paired or unpaired Student’s *t*-test was used to compare two groups and for comparisons of more that 2 groups, ANOVA with Tukey’s or Dunnett’s multiple comparison test was applied. Pearson’s correlation coefficient was used in Figures 4A, 4-figure supple 1A, 5A and 5B. This information is available in Materials and Methods section.

In Figures 2B and 5A, to obtain confidence intervals, p16 or uNK scores at each day of the cycle were fitted into a beta distribution with the fitdistr function from the MASS package in R. The resulting percentile values were smoothed with spline interpolation using the spline function of the basic stats package of R and converted into a heatmap using the Multi Experiment Viewer. Details are provided in Materials and Methods section.

(For large datasets, or papers with a very large number of statistical tests, you may upload a single table file with tests, Ns, etc., with reference to sections in the manuscript.)

**Group allocation**

- Indicate how samples were allocated into experimental groups (in the case of clinical studies, please specify allocation to treatment method); if randomization was used, please also state if restricted randomization was applied
- Indicate if masking was used during group allocation, data collection and/or data analysis

Please outline where this information can be found within the submission (e.g., sections or figure legends), or explain why this information doesn’t apply to your submission:

Our work did not involve a clinical trial or patient randomization.

During the analysis of large patient data sets (figures 2D and 5A), n-numbers in each group (day of menstrual cycle) were dictated by clinic appointment schedules and timing.

Where bias could have influenced experimental outcomes (figure 5B), analysis was blinded where appropriate to maintain fairness. Details of this are included in the Materials and Methods section.

**Additional data files (“source data”)**

- We encourage you to upload relevant additional data files, such as numerical data that are represented as a graph in a figure, or as a summary table
- Where provided, these should be in the most useful format, and they can be uploaded as “Source data” files linked to a main figure or table
- Include model definition files including the full list of parameters used
- Include code used for data analysis (e.g., R, MatLab)
- Avoid stating that data files are “available upon request”

Please indicate the figures or tables for which source data files have been provided:

Source data has been included as excel files for the following figures:

Figure 1. B, D, E and F

Figure 2. A, B and D

Figure 3. A, B, C, D, E, F, G and H

Figure 4. A, C, D, E and F

Figure 5. A, B, D, E and F

Statistical tests used and p-values (where appropriate) are provided.

Details of graph fitting and statistical software packages are provided in Materials and Methods section.
